# Identifying factors predicting prolonged rehabilitation after simultaneous bilateral total knee arthroplasty: a retrospective observational study

**DOI:** 10.1186/s12891-021-04211-x

**Published:** 2021-04-20

**Authors:** Shu Takagawa, Naomi Kobayashi, Yohei Yukizawa, Takayuki Oishi, Masaki Tsuji, Toshihiro Misumi, Yutaka Inaba

**Affiliations:** 1 Department of Orthopaedic Surgery, Yokohama City University Medical Center, 4-57 Urafune-cho, Minami-ku, Yokohama, 232-0024 Japan; 2 Department of Biostatistics, Yokohama City University School of Medicine, 3-9, Fukuura, Kanazawa-ku, Yokohama, 236-0004 Japan; 3 Department of Orthopaedic Surgery, Yokohama City University Graduate School of Medicine, 3-9, Fukuura, Kanazawa-ku, Yokohama, 236-0004 Japan

**Keywords:** Total knee arthroplasty, Rehabilitation, Length of hospital stay, Hemoglobin, Delayed discharge

## Abstract

**Background:**

Rehabilitation is an effective procedure for promoting functional recovery after simultaneous bilateral total knee arthroplasty (TKA); however, it has been cited as a significant economic burden of medical care. We hypothesized that preoperative factors, including age, sex, body mass index, living alone, the knee society function score (KSS), the American society of anesthesiologists (ASA) class, hemoglobin (Hb), albumin level, mean range of motion, and the Kellgren–Lawrence grade, would predict prolonged rehabilitation utilization.

**Methods:**

In total, 191 patients undergoing simultaneous bilateral TKA in a single hospital were enrolled. The successful compliance group included patients who completed their rehabilitation program and could return to their residence within 3 weeks after surgery (*n* = 132), whereas the delayed group included the remaining patients (*n* = 59). Logistic regression analysis was performed using preoperative factors. A prediction scoring system was created using the regression coefficients from the logistic regression model.

**Results:**

Logistic regression analysis revealed that age (β = − 0.0870; *P* <  0.01) and Hb (β = 0.34; *P* <  0.05) were significantly associated with prolonged rehabilitation programs, whereas body mass index, living alone, KSS score, and ASA class were not significantly associated with successful completion of rehabilitation programs; however, these factors contributed to the prediction scoring formula, which was defined as follows:

$$ {\displaystyle \begin{array}{l}\mathrm{Score}=10-\left(0.09\times \mathrm{age}\right)-\left(0.09\times \mathrm{body}\ \mathrm{mass}\ \mathrm{index}\right)-\left(0.56\times \mathrm{living}\ \mathrm{alone}\ \right[\mathrm{alone}:1,\\ {}\mathrm{others}:0\left]\right)+\left(0.03\times \mathrm{KSS}\ \mathrm{stairs}\right)+\left(0.34\times \mathrm{Hb}\right)-\left(1.1\times \mathrm{ASA}\ \mathrm{class}\right).\end{array}} $$

The C-statistic for the scoring system was 0.748 (95% confidence interval, 0.672–0.824). The positive and negative likelihood ratios were 2.228 (95% CI, 1.256–3.950) and 0.386 (95% CI, 0.263–0.566), respectively. These results showed an increase of 15–20% and a decrease of 20–25% in the risk of prolonged rehabilitation. The optimal cutoff point for balancing sensitivity and specificity was 3.5, with 66.6% sensitivity and 78.0% specificity.

**Conclusions:**

Older age and lower preoperative Hb were significantly associated with prolonged rehabilitation programs. We defined a new scoring formula using preoperative patient factors to predict prolonged rehabilitation utilization in patients undergoing simultaneous bilateral TKA.

## Background

Total knee arthroplasty (TKA) is a reliable treatment for damaged knee joints and provides excellent long-term results regarding pain relief and functional restoration in patients with osteoarthritis [1]. As knee osteoarthritis has historically been considered an asymmetric disease [2], contralateral knee osteoarthritis is common in patients with end-stage unilateral osteoarthritis who undergo TKA [3]; therefore, simultaneous bilateral TKA has been commonly performed [4]. Rehabilitation is an effective procedure for promoting functional recovery; however, the average annual rehabilitation cost per patient has increased recently [5].

A previous systematic review has reported on the risk factors for prolonged hospital stay [6], and a few studies have reported on the risk factors for increased duration of rehabilitation utilization [5, 7]. Therefore, predicting the duration of rehabilitation utilization among simultaneous bilateral TKA cases using a preoperative scoring system will be beneficial.

This study aimed to determine the risk factors predicting prolonged rehabilitation utilization after simultaneous bilateral TKA. In addition, we created a new scoring system to predict the necessary duration of physiotherapy after simultaneous bilateral TKA and evaluated the predictive ability of the model.

## Methods

### Description of population

**Fig. 1:**
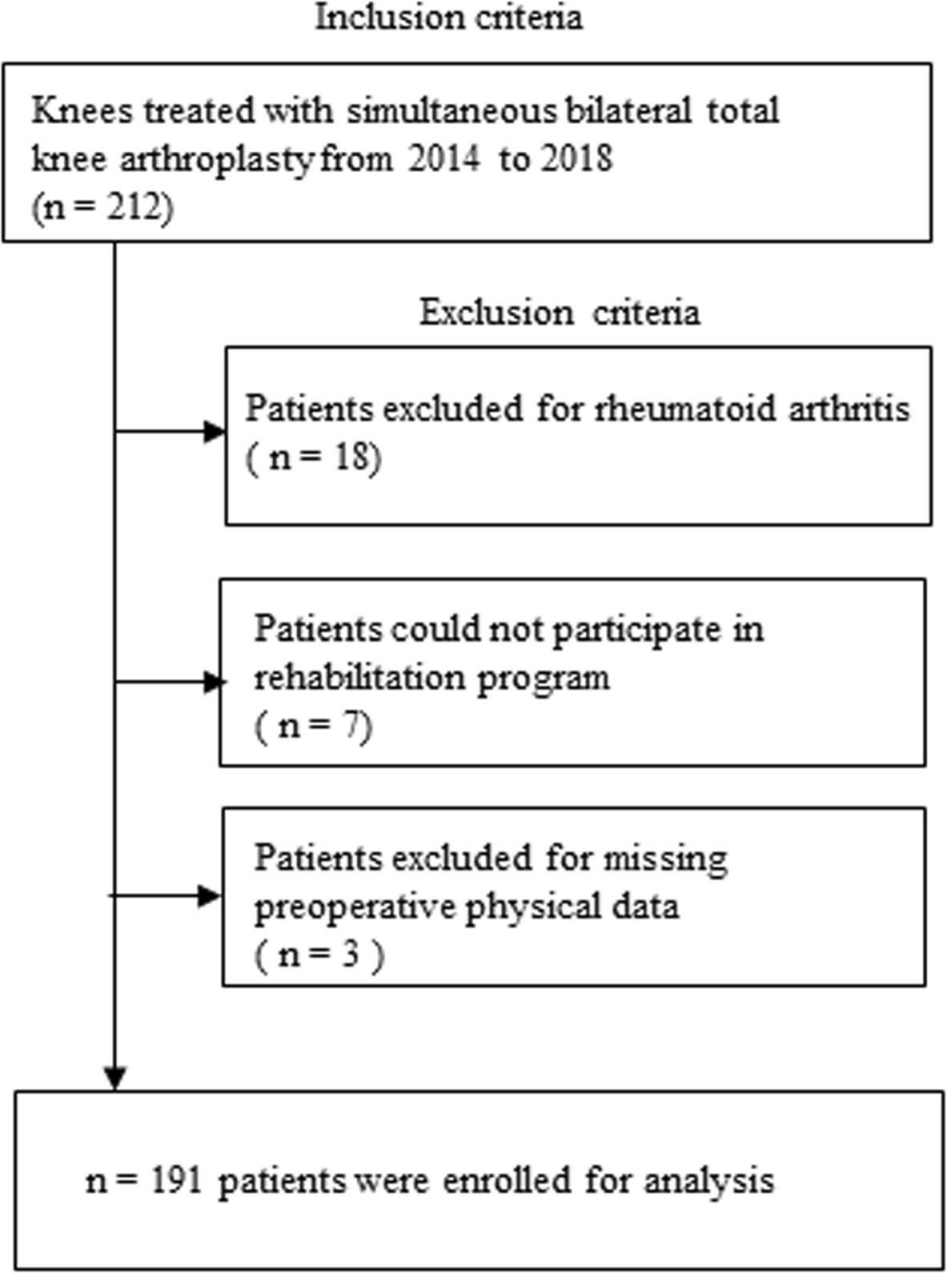
Inclusion and exclusion criteria for the study participants

This retrospective observational study was approved by the Institutional Review Board of Yokohama City University (B190700017), and informed consent was obtained from all patients. Inclusion criteria for the current study included patients whose knees were treated with consecutive, simultaneous bilateral TKA between January 2014 and December 2018 in a single hospital. In total, 212 patients met the inclusion criteria. We excluded 18 patients with rheumatoid arthritis, seven patients who could not participate in the rehabilitation program for reasons, such as postoperative thrombus embolism, and three patients with missing preoperative physical data. Finally, a total of 191 patients were enrolled in the study (Fig. 1). All patients were diagnosed with osteoarthritis.

### Surgical interventions and postoperative treatments

Bilateral TKAs were performed sequentially by one team comprising five experienced orthopedic surgeons. General anesthesia with peripheral nerve block was used for all patients, except for those who did not agree with or could not undergo peripheral nerve block due to complications. A lateral curved longitudinal skin incision and midvastus approach was employed. Intra- and extramedullary techniques were used for placement of femoral and tibial cutting guides, respectively. A modified gap-balancing technique was employed. A tourniquet was inflated before incision and deflated after cement hardening. The Scorpio NRG® posterior-stabilized prostheses (Stryker Orthopedics, Mahwah, NJ, USA) were used in all cases.

All patients, except those with anemia (hemoglobin [Hb] < 11.0 g/dL), underwent two 400 mL autologous blood collections prior to surgery. Postoperative autologous blood transfusion of 800 mL was performed within 3 days after surgery, independent of Hb levels. In patients with Hb level < 11 g/dL, we could not collect autologous blood samples due to our hospital’s autologous blood guidelines, and allogenic blood transfusion was performed in patients with Hb level < 7.5 g/dL or those who were expected to progress to anemia because of postoperative bleeding.

Postoperative pain management comprised non-steroidal anti-inflammatory drugs for internal use with an additional acetaminophen infusion when required. Rehabilitation was started on the day after surgery and continued until discharge. Full weight-bearing was allowed from the day after the surgery. Patients underwent a stepwise program to achieve the ability to use a wheelchair, followed by walking using a walker and T-cane, depending on compliance. In our facility, the rehabilitation goal was set within 3 weeks post-surgery following the clinical pathway. Generally, patients could not be discharged on time if stable independent walking with a T-cane and stepping up and down the stairs were not achieved within 3 weeks postoperatively. Patients were informed that a hospital transfer would be required if rehabilitation programs needed more than 3 weeks. Patients were discharged to their homes when they were able to walk using a T-cane and step up and down the stairs independently.

### Data collection

Patient data, including age, sex, body mass index (BMI), living alone, Knee Society function score (KSS) [8], American Society of Anesthesiologists (ASA) class, preoperative Hb levels (g/dL), serum albumin (Alb) level (g/dL, measured 8 weeks prior to surgery before autologous blood collection), mean range of motion (average of the two sides), and mean Kellgren–Lawrence classification (K–L grade, average of the two sides), were collected from the hospital’s computer databases [9]. All parameters were obtained from the medical records at the time of preoperative outpatient examination and through patient interviews. The rehabilitation program utilization was calculated as the duration from the date of operation to the date of discharge from the hospital to the patient’s home. Patients who were transferred to other hospitals due to delayed rehabilitation were analyzed separately as transferred cases. Patients who could return to their residence within 3 weeks after surgery were assigned to the compliance group (*n* = 132), whereas those who could not and transferred patients were assigned to the delayed group (*n* = 59).

### Statistical analysis

Values are expressed as mean ± standard deviation (SD). Compliance and delayed groups were defined as dependent variables. Age, sex, BMI, living alone, KSS, the ASA class, Hb levels, Alb levels, mean range of motion, and the K–L grade were defined as predictor variables. Normally distributed data were analyzed using a non-paired t-test, whereas non-normally distributed data were analyzed using the Mann–Whitney U test; the chi-square test was used for between-group comparisons of the preoperative parameters. Multivariate logistic regression analysis using a forward-backward stepwise selection method was performed to determine factors influencing successful compliance or prolonged rehabilitation programs. Standardized regression coefficients (β) and associated *P*-values were determined to assess statistical significance. P-value < 0.05 was considered statistically significant. According to the methods of Sullivan et al. [10] and Oba et al. [11], a prediction scoring formula was created using the multivariate logistic regression model results. Steyerberg et al. reported that stepwise selection with low alpha (*P* <  0.05) leads to a relatively poor model performance in cases with small samples [12]. Therefore, we created a prediction formula using factors resulting from the regression analysis (*P* <  0.20). Similarly, Zemek et al. selected variables using factors with *P* < 0.20, which was considered to have some correlation with the results [13].

Scores were calculated by adding the results of multiplications between each independent factor and its coefficient β. To simplify the formula, up to three significant digits of the coefficient β were used. Ten points were added to the formula to make the score positive. C-statistics and likelihood ratio were used to assess the predictive ability of the model. Patients were stratified according to their score, and the observed probability of being in the compliance or delayed group was assessed. All statistical analyses were performed using the JMP 12.2 software (SAS Institute Inc., Cary, NC, USA).

## Results

**Fig. 2:**
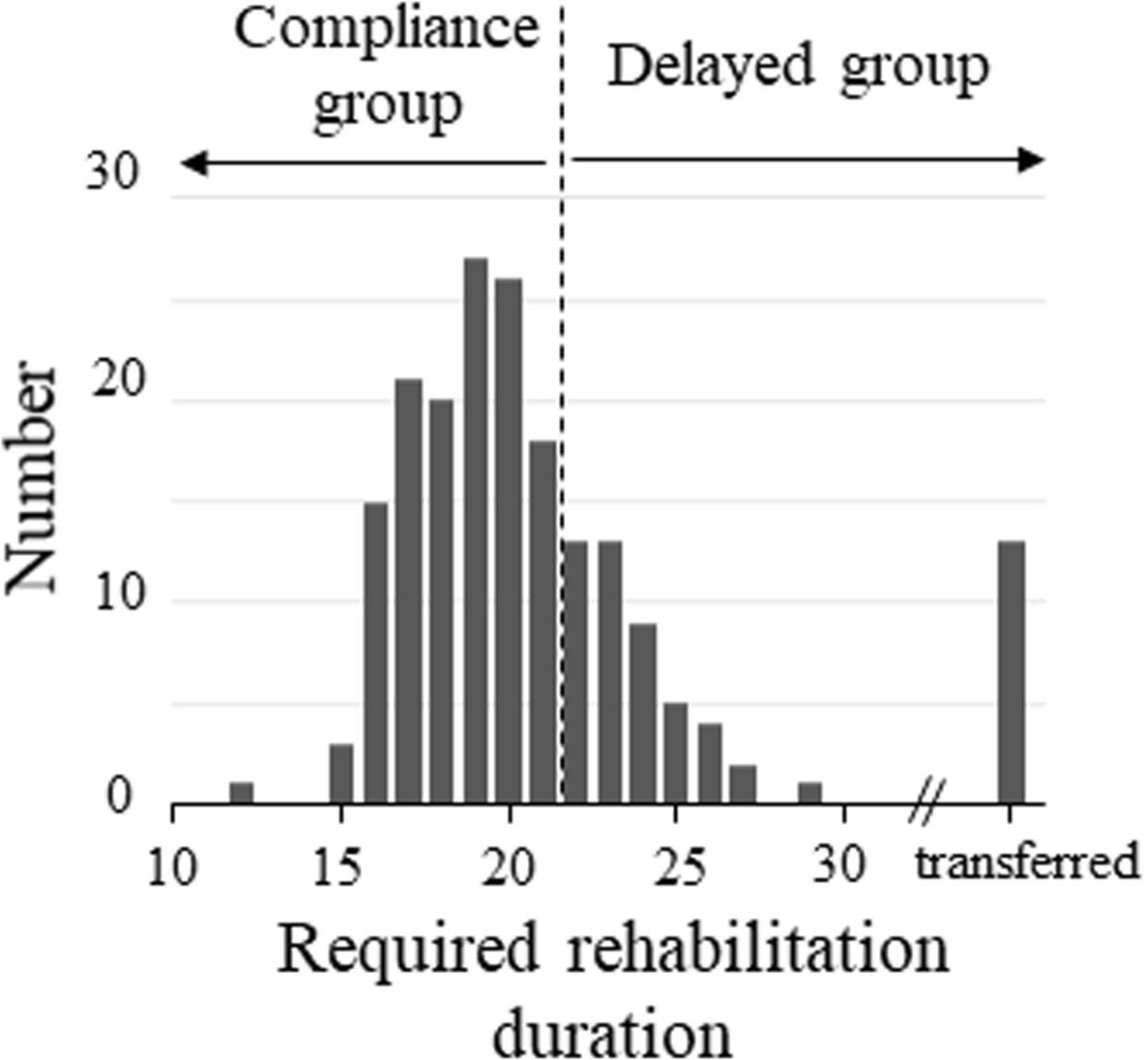
Distribution of the required rehabilitation durations. Patients who required a rehabilitation duration of less than 21 days after surgery were assigned to the compliance group. Patients with a required rehabilitation duration of over 21 days and transferred cases were assigned to the delayed group

The distribution of the required rehabilitation duration is shown in Fig. 2. The range of duration of the compliance and delayed groups was 12–21 and 22–29 days, respectively (mean 18.5 and 23.6 days, respectively). The mean duration of the delayed group excluded values from 13 transferred cases.

Patients in the delayed group were significantly older, used more walking aids, and had a higher ASA class as well as lower Hb and Alb levels than those in the compliance group. No significant differences between the groups were observed in sex ratio, BMI, the incidence of living alone, walking ability, stair-climbing ability, mean range of motion, and mean K–L grade. The delayed group included 13 transferred cases. Patients’ data are summarized in Table 1. Table 2 shows the results of the logistic regression analysis, which indicated that age (β = − 0.0870; *P* = 0.0059) and Hb (β = 0.340; *P* = 0.0370) were significantly associated with rehabilitation duration.

**Table 1:** Summary of patient data

All patients (*n* = 191)	Compliance group (*n* = 132)	Delayed group (*n* = 59)	*P*-value
**Age, years**			
< 60	4	1	
60–70	26	6	
70–80	76	25	
80–90	26	27	
Average	74.2	77.9	< 0.001^a^
**Female, %**	83.3%	88.1%	0.393^b^
**Body mass index, kg/m**^**2**^			
< 20	3	0	
20–25	38	20	
25–30	66	22	
30–35	22	13	
> 35	3	4	
Average	27.1	27.7	0.706 ^c^
**Living alone, %**	15.9%	27.1%	0.077^b^
**Knee Society score**			
Walking	24.5	22.7	0.138 ^c^
Stairs	27.1	23.2	0.061 ^c^
Walking aids used	3.5	5.0	0.043 ^c^
Total	48.1	41.6	0.057 ^c^
ASA class			
I	11	2	
II	119	51	
III	2	6	
Average	1.93	2.07	0.009 ^c^
**Hemoglobin, g/dL**	13.1	12.6	0.014^a^
**Albumin, g/dL**	4.4	4.3	0.018^a^
**Mean range of motion**			
Extension	12.7	12.3	0.704 ^c^
Flexion	113.4	114.5	0.964 ^c^
**Mean K–L grade**	3.86	3.89	0.753 ^c^
**Length of stay (days, excluding transferred cases)**	18.5	23.6	< 0.001 ^c^
**Transferred cases, number**	0	13	

Values are presented as mean. *P*-values were obtained using the Mann–Whitney U test or chi-square test

*n* number, *ASA* American society of anesthesiologists, *K–L grade* Kellgren–Lawrence classification

^a^Non-paired t-test

^b^chi-square test

^c^Mann–Whitney U test

**Table 2:** Results of regression analysis

Variable	Β	*P*-value	Odds ratio	CI (95%)	
				Lower	Upper
**Age**	−0.0870	0.00590	1.09	1.03	1.16
**BMI**	−0.0892	0.0565	1.09	0.998	1.20
**Living alone**	−0.563	0.165	0.579	0.257	1.27
**Knee Society score: stairs**	0.0267	0.140	0.978	0.948231	1.01
**Hemoglobin, g/dL**	0.340	0.037	0.712	0.511493	0.971
**ASA class**	−1.13	0.0724	3.09	0.964960	12.0

*CI* confidence interval, *BMI* body mass index, *ASA* American society of anesthesiologists

The prediction scoring formula was created as follows: score = 10 – (0.09 × age) – (0.09 × BMI) – (0.56 × living alone [alone: 1, others; 0]) + (0.03 × KSS stairs) + (0.34 × Hb) – (1.1 × ASA class). Table 3 shows the formula and the observed probability of being in the compliance group for scores of 0–7 points.

**Table 3:** Scoring formula and the observed probability of required rehabilitation duration

Scoring formula		Outcomes				
Factors	Points	Total score	n	Compliance group	Delayed group	Observed probability of required rehabilitation duration conformance, %
	+ 10	0–1	1	0	1	0.0%
Age, (years)	× −0.09	1–2	9	2	7	22.2%
BMI	× −0.09	2–3	40	20	20	50.0%
Living alone	× −0.56	3–4	74	52	22	70.3%
KSS: stairs	× 0.03	4–5	47	41	6	87.2%
Hemoglobin, g/dL	× 0.34	5–6	13	11	2	84.6%
ASA class	× −1.1	6–7	7	6	1	85.7%

*n* number, *KSS* knee society score, *BMI* body mass index

**Fig. 3:**
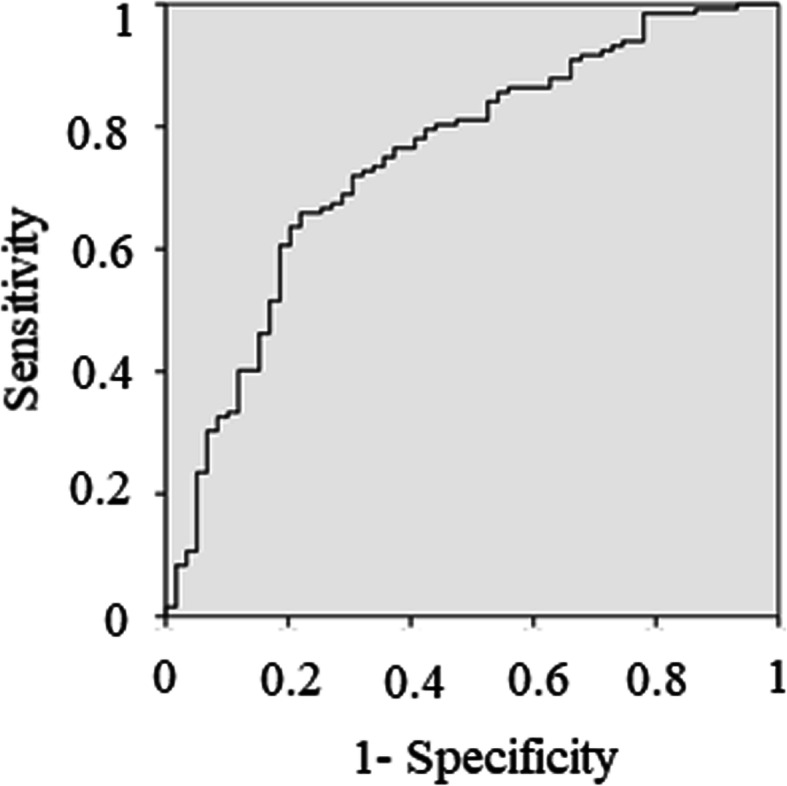
Prediction of compliance with the target. The figure depicts the prediction of compliance with a target of less than 3 weeks duration of required rehabilitation

Figure 3 shows the receiver operating characteristic curve of the scoring system. The area under the curve (AUC) was 0.748 (95% confidence interval [CI], 0.672–0.824). The optimal cutoff point for balancing sensitivity and specificity was 3.5, with 66.6% sensitivity and 78.0% specificity. Positive likelihood ratio was 2.228 (95% CI, 1.256–3.950), and negative likelihood ratio was 0.386 (95% CI, 0.263–0.566).

## Discussion

The main point of clinical relevance in this study is the ability of preoperative factors to predict the likelihood of prolonged rehabilitation after simultaneous bilateral TKA. Based on our scoring formula, we predicted that less than half of the patients with a preoperative score of < 3 points would not complete the rehabilitation programs and would not be able to return to their residence.

The role of rehabilitation programs is considered essential as patients rapidly recover independent movement and transfer capacities during this period; thus, contributing to an early home discharge [7]. The mean length of stay (LOS) after TKA procedures has gradually decreased from 15 days to 5 days over the last few decades in Europe and the United States [6]; however, long-term hospitalization remains the norm in Japan [14]. In Japan, LOS after TKA included rehabilitation programs. The cutoff value was defined as 3 weeks in this study. The first reason for this is that the Japanese insurance system (diagnosis procedure combination [DPC]) defined 22 days post-hospital admission as the target to proceed with TKA. Thus, the Japanese insurance system enforces the LOS goal within 3 weeks post-TKA as the acceptable clinical pathway. Second, in the early postoperative phase, the quadriceps strength is significantly lower than the preoperative levels [15], and it takes approximately 3 weeks to restore lower extremity function to pre-surgery levels [16].

Older age was associated with decreased complication functional scores (as represented by the KSS score) and increased postoperative complication rates [17]. Blood management has the most significant impact on LOS after TKA, and correction of preoperative anemia with iron and/or erythropoiesis-stimulating agents helps in decreasing transfusion risk [18]. Hb level is a factor that can be improved preoperatively. Therefore, it is important to inform the medical team that preoperative care of anemia using drugs and/or daily life guidance might reduce the required duration of rehabilitation postoperatively. In accordance with past findings, our study identified older age and low preoperative Hb levels as factors with the strongest association with delayed achievement of rehabilitation programs after simultaneous bilateral TKA. In contrast, BMI, living alone, KSS score, and ASA class were not significantly associated with the achievement of rehabilitation programs but contributed to the prediction scoring formula.

There are no obvious clinical indicators of the duration of rehabilitation required for functional recovery after simultaneous bilateral TKA. LOS following TKA decreases yearly [6], and it varied among patients who did not need a rehabilitation program after discharge. Physiotherapy exercises following TKA have both short- and long-term benefits for patients [19]. Because of limited medical resources, preoperative assessment of each patient is required to estimate the rehabilitation period needed for them to become independent and capable of activities of daily living (ADL); such assessment may improve the efficiency of rehabilitation programs. Therefore, the identification of individual predictive factors will benefit clinical decision-making. Quadriceps muscle strength is an important determinant of physical function after TKA [6]. Therefore, lower limb strengthening may be effective in achieving independence to complete ADL earlier. Recently, Ueyama et al. [20] reported that nutritional supplements rich in essential amino acids prevent femoris muscle atrophy and postoperatively accelerate the time taken to recover ADL to that of baseline level. If the preoperative prediction system can identify patients with delayed recovery of ADL, it may be possible to selectively and efficiently perform interventions, such as nutritional supplementation.

Return to residence after surgery is associated with multiple factors, including the patient’s social background, which may influence the achievement of rehabilitation programs. Our study demonstrated a simple method to calculate scores at the time of scheduling outpatients for surgery. The prediction scoring system may facilitate social services when early discharge is likely, and adjustment when transfer to a rehabilitation hospital is likely. For cases with lower scores under the 3.5-point cutoff value, it is especially important to treat anemia and inform patients and their families that it might take longer to perform ADL independently.

The purpose of this investigation was to derive a prediction model to identify patients at risk for prolonged rehabilitation following simultaneous bilateral TKA. Further investigation is required to validate the model and more data must be added to refine the model. We restricted this investigation to patients undergoing bilateral TKA within the Japanese healthcare system. While postoperative care varies among countries and regions, our main findings in which age and low Hb predict longer recovery are likely generalizable beyond these restrictions. We chose to construct the prediction formula using factors with *P* < 0.20. In cases wherein only age and Hb values were used (*P* < 0.05), the AUC was 0.697 (95% CI, 0.603–0.786). Hence, we included factors with P < 0.20 for the construction of the prediction formula to avoid a relatively poor model performance, which may result from using only factors with *P* < 0.05. In addition, we recognize that there may be other factors that contribute to the prediction of postoperative outcomes. Preoperative pain assessment was not included in this study. Preoperative central sensitization was recently identified as a significant risk factor for postoperative persistent pain and dysfunction [21], leading to prolonged duration of rehabilitation. Thus, preoperative central sensitization might be a significant factor influencing the scoring formula and is suggested for inclusion in future investigations.

Several limitations of our study should be addressed. First, the sample size in this study was relatively small. Therefore, further studies with a larger sample size are required to validate the reliability of our scoring system. Second, we excluded patients with rheumatoid arthritis and postoperative complications. Patients with rheumatoid arthritis tend to have a longer duration of hospitalization and discharge to rehabilitation facilities than patients with osteoarthritis, independent of adverse events [22]. Third, the study population predominantly comprised Japanese, and the results may not be generalizable to other demographic groups. In other countries, LOS following TKA is much shorter than that in Japan. Furthermore, LOS was strongly influenced by the patient’s expectation of when they could be discharged home based on preoperative education programs [23]. Hence, additional investigations are needed to validate the scoring system in other countries and different situations. Fourth, the a positive likelihood ratio between 2 and 3 indicated an increase in probability by 15–20%, and a negative likelihood ratio between 0.3 and 0.4 indicated a decrease in probability by 20–25% [24]. These results did not show a significant increase and/or decrease in the likelihood of predicting increased rehabilitation duration. Fifth, age was the most influential factor in this study; therefore, other risk factors might not be fully identified. To create a more clinically useful predictive formula, it might be useful to perform analysis in a certain range of older age group. Therefore, to construct a more accurate prediction formula, it is necessary to collect and examine a larger number of cases and additional information before applying these predictors in actual clinical practice.

## Conclusion

Older age and lower preoperative Hb were significantly associated with prolonged rehabilitation programs. We defined a new scoring formula using preoperative patient factors to predict prolonged rehabilitation utilization in patients undergoing simultaneous bilateral TKA. Further studies are warranted to confirm the value of this scoring system and improve its accuracy.

## Data Availability

The datasets during and/or analyzed during the current study available from the corresponding author on reasonable request.

## Abbreviations

TKA: Total knee arthroplasty
KSS: Knee society function score
ASA: American society of anesthesiologist
BMI: Body mass index
Hb: Hemoglobin
Alb: Albumin
LOS: Length of stay
DPC: Diagnosis procedure combination
K–L: Kellgren–Lawrence classification
ADL: Activities of daily living
